# Strain identity effects contribute more to *Pseudomonas* community functioning than strain interactions

**DOI:** 10.1093/ismejo/wraf025

**Published:** 2025-02-08

**Authors:** Jos Kramer, Simon Maréchal, Alexandre R T Figueiredo, Rolf Kümmerli

**Affiliations:** Department of Quantitative Biomedicine, University of Zurich, Winterthurerstrasse 190, 8057 Zurich, Switzerland; Department of Environmental Systems Sciences, ETH Zurich, Universitätsstrasse 16, 8092 Zurich, Switzerland; Department of Quantitative Biomedicine, University of Zurich, Winterthurerstrasse 190, 8057 Zurich, Switzerland; Department of Quantitative Biomedicine, University of Zurich, Winterthurerstrasse 190, 8057 Zurich, Switzerland; Department of Biology, University of Oxford, 11a Mansfield Road OX1 3SZ, Oxford, United Kingdom; Department of Quantitative Biomedicine, University of Zurich, Winterthurerstrasse 190, 8057 Zurich, Switzerland

**Keywords:** Siderophores, Pyoverdine, *Pseudomonas,* Social interactions, Social behavior, Community functioning, Community productivity, Microbiome engineering

## Abstract

Microbial communities can shape key ecological services, but the determinants of their functioning often remain little understood. While traditional research predominantly focuses on effects related to species identity (community composition and species richness), recent work increasingly explores the impact of species interactions on community functioning. Here, we conducted experiments with replicated small communities of *Pseudomonas* bacteria to quantify the relative importance of strain identity versus interaction effects on two important functions, community productivity and siderophore production. By combining supernatant and competition assays with an established linear model method, we show that both factors have significant effects on functioning, but identity effects generally outweigh strain interaction effects. These results hold irrespective of whether strain interactions are inferred statistically or approximated experimentally. Our results have implications for microbiome engineering, as the success of approaches aiming to induce beneficial (probiotic) strain interactions will be sensitive to strain identity effects in many communities.

## Introduction

Microbial communities are present in almost all natural and anthropogenic environments on earth, and shape important ecological services from primary production to the decomposition of organic matter and the fixation of greenhouse gases [1, 2]. Given that many of these services can be harnessed for biotechnological applications such as bioremediation and the production of biofuels [3–6], recent years have seen a massive surge in studies investigating how contributions of specific microbial players and interactions between players affect functions (collective properties) of microbial communities [7–12]. This research has elucidated the independent effects of various factors such as community composition and species richness on key functions including productivity, nutrient cycling, and invasion resistance. By contrast, the combined influence of these factors is less well understood although capturing these integrative effects seems crucial to predicting and manipulating how microbial communities work [13, 14].

Traditionally, studies on factors shaping community functioning mainly focus on community composition and species richness [7, 15–17]. Community composition typically matters because some species may contribute more to functioning than others [7, 17]. Conversely, species richness typically matters because higher numbers of species increase the likelihood of including species with different niche requirements or strong effects on functioning [7, 15]. In both cases, effects on community functioning are ultimately driven by characteristics inherent to specific community members (identity effects). More recently, research has increasingly focused on effects on functioning driven by interactions among community members (interaction effects) [8, 10, 11, 13, 18–20]. In microbes, these interactions are often mediated by secreted products – such as toxins, antibiotic-degrading enzymes, and iron-scavenging siderophores – and can have strong effects on community dynamics [21–23]. Although such interactions are clearly relevant, it remains unclear how their impact on functioning compares to the impact of identity effects because studies typically focus on only one of the two factors [24, 25].

Here, we tackle this knowledge gap by comparing the impact of strain identity and strain interactions on the functioning of small communities of pseudomonads. *Pseudomonas* is a diverse and widespread genus of γ-proteobacteria, occurring in soil, freshwater, and host ecosystems [26]. Pseudomonads produce and interact through a range of secreted compounds including proteases, biosurfactants, and the fluorescent siderophore pyoverdine [27, 28]. This versatility has long made representatives such as *P. aeruginosa* and *P. fluorescens* important models for studying microbial interactions [29–32]. More recently, pseudomonads have increasingly been used to investigate how social traits affect community functioning, including productivity, plant protection, and invasion resistance [8, 9, 33–35].

In our study, we used 64 diverse soil and freshwater *Pseudomonas* strains belonging to four phenotype classes to examine how strain identity and strain interactions jointly shape two community functions: productivity and the production of iron-scavenging pyoverdines. Pyoverdines are a diverse group of siderophores, and each specific pyoverdine can either promote the growth of strains possessing matching uptake receptors or inhibit the growth of strains without these receptors [36]. To quantify the positive and negative interactions through pyoverdines and other secreted compounds, we conducted supernatant feeding assays under different conditions. These assays allowed us to calculate community-level interaction metrics. Next, we grew all strains in monocultures and all combinations of two, three, or all four strains per community, and measured their productivity and pyoverdine production over time. This allowed us to leverage an established linear model method [24] to statistically disentangle the effects of strain identity and strain interactions, and to compare the numerically derived measure of strain interactions with our experimentally measured interaction metrics. Overall, our results show that although strain interactions can significantly affect the functioning of *Pseudomonas* communities, their effects are generally outweighed by those of strain identity.

## Materials and methods

### Strain selection

We drew strains from an established collection of 315 pseudomonads, isolated from eight soil and eight freshwater (pond) samples (18–20 isolates per sample). Sampling, phenotyping and genetic identification of these strains are described elsewhere [27, 28, 37]. Here, we selected a subset of 64 strains, including four strains from each of the 16 samples [hereafter: community], based on their ability to produce pyoverdine and exo-proteases [28]. Our experiments focused on pyoverdine as the primary phenotype of interest and contrasted conditions where it is important for growth with conditions where it is not. Conversely, we included protease production as a secondary phenotype of interest to increase the likelihood of including phenotypically and phylogenetically diverse strains (Fig. S1). Specifically, we chose per community (i) one strain producing pyoverdine and proteases, (ii) one strain producing only pyoverdine, (iii) one strain producing only proteases, and (iv) one strain producing neither pyoverdine nor proteases (supplementary methods, Fig. S2). Each community thus featured two strains producing pyoverdine at high levels (the double and the pyoverdine producer; hereafter producers PVD_PRO_ and PVD) and two strains producing no to little pyoverdine (the protease and the non-producer; hereafter non-producers NON_PRO_ and NON). Phylogenetic analyses based on partial *rpoD* sequences confirmed for all but one community that the four selected strains belonged to distinct phylogenetic clades (Fig. S1, Table S1). Hereafter, we use “strain type” to refer to the four phenotype classes described above and “strain ID” to refer to individual representatives of our 64 strains. This strain selection procedure resulted in 16 replicated communities that similarly differ in phenotype composition and phylogenetic diversity.

### Growth and siderophore production measurements

We quantified growth and pyoverdine production of all strains under iron-limited and iron-rich conditions. First, we grew precultures in 24-well plates with 1.5 ml lysogeny broth (LB, 2% w/v) per well under static conditions for 48 h. Subsequently, we washed cells in 0.85% NaCl and measured their growth ([OD_600_]; optical density at 600 nm) against a 0.85% NaCl blank using an Infinite M200 PRO microplate reader (Tecan, Männedorf, Switzerland). Next, we adjusted precultures to OD_600_ = 0.4 and inoculated 2 μL of each adjusted culture into 96-well plates containing 200 μL medium per well in fourfold replication. We used two variants of CAA medium (5 g casamino acids, 1.18 g K_2_HPO_4_·3H_2_O, and 0.25 g MgSO_4_·7H_2_O per liter), a medium traditionally used to study the production and effects of pyoverdine in pseudomonads [38]. Specifically, we used an iron-limited variant supplemented with 25 mM HEPES buffer, 20 mM NaHCO_3_ and 100 μg/mL apo-transferrin (a strong iron-chelator), and an iron-rich variant supplemented with 25 mM HEPES buffer and 40 μM FeCl_3_. After 24 h of static incubation at 28°C, we quantified growth [OD_600_] and pyoverdine production ([RFU_pvd_]; relative fluorescence units; excitation|emission at 400|460 nm) after 120 s of vigorous shaking using the microplate reader.

### Supernatant assay

We explored interactions through secreted compounds under iron-limited and iron-rich conditions by exposing each strain to its own supernatant and to each supernatant from its community members. We harvested supernatants from cultures grown in the above-described experiment by spinning them through 96-well filter plates with a 3 μm glass fiber/0.2 μm Supor membrane (AcroPrep Advance; Pall Corporation, Port Washington, USA) and then collecting the sterile supernatants in 96-well plates. These plates were sealed and stored at −20°C until further use. Next, we grew precultures of all strains, washed and adjusted them as before, and subjected them to three treatments: (i) SN_limited_: 180 μL of iron-limited CAA supplemented with 20 μL of supernatant generated under iron-limited conditions; (ii) SN_rich_: 180 μL of iron-rich CAA supplemented with 20 μL of supernatant generated under iron-rich conditions; and (iii) SN_control_: 180 μL of iron-limited or iron-rich CAA supplemented with 20 μL of 0.85% NaCl (mimicking spent medium). Strains were grown in threefold (SN_control_) or fourfold (SN_limited_ and SN_rich_) replication. We measured growth [OD_600_] and pyoverdine production [RFU_pvd_] of each replicate after 24 h and 48 h of incubation at 28°C under static conditions. We calculated the effects of each supernatant on all four community members as growth effects: GE_treatment_ = (SN_treatment_/SN_control_), where SN_treatment_ = SN_limited_ or SN_rich_, with growth values being calculated as the median growth across replicates. Values smaller and greater than one indicate growth inhibition and stimulation, respectively.

We calculated three summary measures of supernatant-based interactions for each possible combination of two, three or all four strains per community. We calculated (i) the mean absolute effect and (ii) the proportion of positive effects to separately capture the strength and sign of supernatant effects. We then calculated (iii) an “interaction score”, which incorporates information on both strength and sign. We first classified for each donor-receiver pair whether their reciprocal supernatant effects were positive, negative, or neutral (i.e., whether SN_treatment_ values differed from SN_control_ values; Table S2), resulting in six possible pairwise interaction types: mutual stimulation [+/+], one-way stimulation [+/0], no effect [0/0], contrasting effects [+/−], one-way inhibition [0/−], and mutual inhibition [−/−]. Finally, we calculated interaction scores by first assigning a value of +1, 0, and − 1 for stimulatory, neutral, and inhibitory effects, respectively, and then calculating the average score across all interactions occurring in each two-, three-, and four-strain community. Interaction scores smaller and greater than zero indicate that inhibitory and stimulatory interactions prevail, respectively.

### Competitions

To be able to assess the effects of strain interactions and strain ID on community functioning, we competed each strain against one, two, or all three other strains of its community under iron-limited and iron-rich conditions, and quantified community productivity and total pyoverdine production over time. We included monocultures as controls, resulting in 15 cultures per community (4x1 strain +6x2 strains +4x3 strains +1x4 strains). We grew precultures from freezer stocks in 50 ml Falcon tubes containing 5 mL LB at 28°C under shaking conditions (170 rpm). After 48 h of incubation, we washed cells in 0.85% NaCl, measured OD_600_ of each culture against a 0.85% NaCl blank, and adjusted strains to OD_600_ = 0.2. Next, we assembled the mixes and inoculated them at OD_600_ = 0.01 starting density in either 6-fold (4-strain competitions) or 5-fold (other conditions) replication into 96-well plates containing 190 μL of iron-limited or iron-rich medium per well. We used a substitutive design: overall starting density remains constant across different mixes, whereas individual strain density decreases when strain richness increases [8, 39]. We incubated plates in a plate reader at 28°C under static conditions and measured the productivity [OD_600_] and total pyoverdine production [RFU_pvd_] of each culture every 15 min over 48 h. We calculated integrals of productivity and total pyoverdine production as our primary measures of community functioning. For some analyses, we additionally calculated deviations from expected productivity and pyoverdine production as DEV_trait_ = TV_mix_ – mean(TV_mono_), where TV_mono_ = trait values of monocultures of strains in the mix. DEV_trait_ indicates whether the trait value (productivity or pyoverdine production) of a specific strain mix is lower or higher than expected based on the trait values observed for monocultures of the constituent strains.

### Linear model method

To compare the effects of strain ID and strain interactions on community functioning, we used a previously developed linear model (LM) method that is frequently used (e.g., [39–42]) to partition the variance in a community-level trait between different factors of interest [24] (see Fig. S3 for a graphical representation). This method uses a series of three LMs to sequentially account for (i) the influence of strain richness (entered as a continuous variable), (ii) strain ID (entered as the presence [categorical] of a particular strain in a particular strain combination), and (iii) statistical strain interactions (strain richness, entered as categorical variable). Whereas entering strain richness as continuous variable accounts for a linear increase of functioning with strain richness, its subsequent entry as a categorical variable tests for nonlinear (non-additive) effects, thereby providing a measure of between-strain interactions [24]. The first LM uses the focal trait of interest as a response, whereas subsequent LMs are fitted on the residuals extracted from the respective previous model. Effects obtained for strain ID and strain interactions are orthogonal and thus independent of the order in which the corresponding LMs are fitted [24]. We ran LMs separately for each community and experimental condition on data from all 11 combinations of two or more strains, focusing on community-level productivity and pyoverdine production as our primary traits of interest. When examining the contributions of different strain types to community functioning, we included DEV_productivity_ and DEV_pyoverdine_ as additional traits to identify strain types driving positive or negative deviations of functioning from expectation. To examine the relative importance of strain interactions and strain ID, we extracted the mean squares from the relevant LMs [ii + iii] [24]. Given that non-linear richness terms provide a purely statistical proxy for strain interactions, we additionally considered our supernatant-based interaction measures. To this end, we replaced the strain richness term in the last LMs [iii] by either the interaction score or the mean absolute supernatant effect and the ratio of positive supernatant effects. We then extracted the corresponding mean squares and included them together with the mean squares for strain ID and non-linear strain richness in an across-community comparison (see below). To examine whether specific strain types contributed disproportionally to community functioning, we extracted the linear model coefficients obtained for each strain from the LMs [ii] focusing on strain ID. These coefficients provide a measure of each strain’s effect on functioning relative to that of the average in the community [24].

### Statistical analysis

We first tested whether growth or pyoverdine production differed between strain types (PVD_PRO_, PVD, NON_PRO_, or NON) and conditions (iron-rich or iron-limited). Next, we tested whether supernatant effects (GE_treatment_) differed between conditions or supernatant donor and receiver types. To examine the relative importance of strain interactions and strain ID on productivity and pyoverdine production, we tested for differences between the mean square values obtained from our LM decomposition for strain ID, non-linear strain richness, the interaction score, and the combination of mean absolute supernatant effect and the ratio of positive effects (see above), accounting for differences between conditions. Similarly, we examined the contributions of different strain types to (deviations from expected) community functioning by comparing the strain coefficients obtained through the LM method across our communities. To examine whether strain interactions consistently shape functioning across communities, we finally tested whether deviations from expected productivity (DEV_productivity_) were shaped by strain richness (2, 3, or 4; categorical), condition, or the interaction score summarizing supernatant-based interactions in the different sets of strains. We always included the strains’ habitat-of-origin (soil or freshwater) as a co-factor into our models, as the habitat is an inherent part of our study design. However, we do not report the corresponding results here because they do not affect our main findings and were rarely significant (all results are reported in the supplementary material).

We implemented our analyses in R 4.4.1 (www.r-project.org) using LMs, generalized least squares (GLS) models, and linear mixed models (LMMs). GLS models and LMMs were implemented using the *gls* and *lme* functions (nlme package) [43]. We obtained *P*-values of effects in these models using the *Anova* function (car package) [44]. We used the emmeans package [45] for post hoc analyses and adjusted *P*-values for multiple testing (n_test_ > 2) using the false discovery rate. Unless stated otherwise, models were initially fitted with all possible interaction terms. Where required, we transformed response variables to obtain normally distributed residuals. To account for the non-independence of strains from the same community and for multiple measurements of each strain under different conditions, we initially fitted all models as random intercept models using community and, in case of repeated measurements, strain (mixture) identity nested within community as random effect(s). To select final models, we first used the Akaike information criterion (AIC) to simplify the random effect structure and to select an appropriate variance structure (using the weights-argument in the *gls* and *lme* function) where residual plots indicated a deviation from homogeneity [46]. Subsequently, we simplified the fixed component by dropping non-significant interaction terms (*P* > 0.05). All final models are detailed in Table S3.

## Results

### Growth and pyoverdine production profiles of *pseudomonas* strains

**Figure 1:**
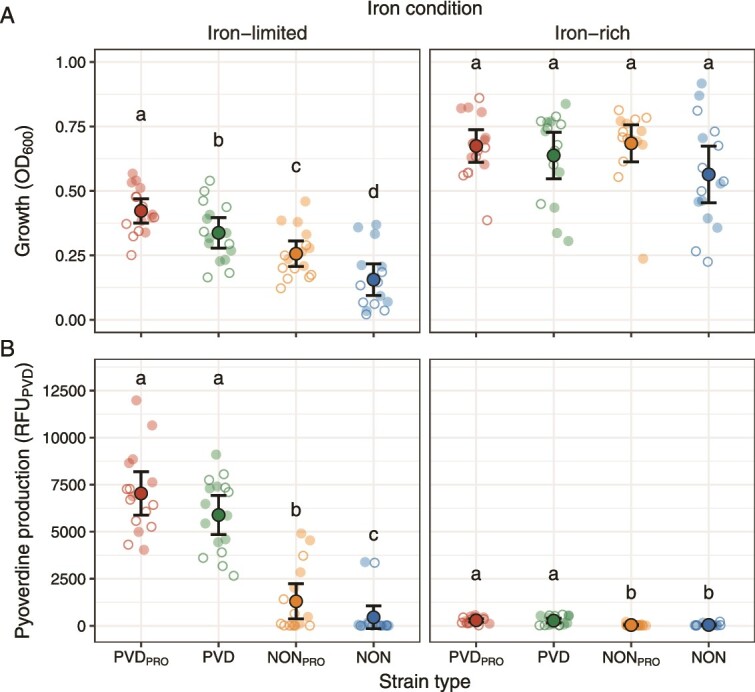
**Strain types differ in their growth and pyoverdine production profiles.** (A) Growth and (B) pyoverdine production of PVD_PRO_, PVD, NON_PRO_, and NON strains isolated from eight soil (empty small circles) and eight freshwater (filled small circles) communities (one strain per type and community), measured under iron-limited and iron-rich conditions. Small circles represent the median of four replicates obtained for each strain under each condition. Large circles and black lines show mean and confidence interval, respectively. Letters show significantly different types in each iron conditions. All types grew more and, with the exception of NON strains, produced less pyoverdine under iron-rich as compared to iron-limited conditions (detailed statistical results are provided in Table S5).

We first confirmed that pyoverdine producers (PVD_PRO_ and PVD) and non-producers (NON_PRO_ and NON) behaved as expected by quantifying pyoverdine production and growth under iron-limited and iron-rich conditions. As expected, we found that the producer types featured higher pyoverdine production than the non-producer types and that pyoverdine favors growth when iron is scarce (Table S4, S5, Fig. 1). Specifically, PVD_PRO_ and PVD grew better than the non-producers under iron limitation, whereas NON_PRO_ reached higher densities than NON. The growth differences between PVD and NON_PRO_ were small despite marked differences in pyoverdine production (Fig. 1A, B), suggesting that NON_PRO_ might produce secondary siderophores such as pyochelin or yersiniabactin [36]. Indeed, when measuring the total iron-chelating activity using the cholorimetric CAS assay [47], we found that NON_PRO_ featured similar activity as PVD (supplementary material; Table S6, Fig. S4). Under iron-rich conditions where siderophores are not required for sustained growth, all strain types reached similar densities (Table S5A, Fig. 1A) and produced little pyoverdine (iron-rich vs. iron-limited: PVD_PRO_: *t*_70_ = −14.20, *P* < 0.001; PVD: *t*_70_ = −12.98, *P* < 0.001; NON_PRO_: *t*_70_ = −4.35, *P* < 0.001; NON: *t*_70_ = −1.19, *P* = 0.239, Fig. 1B).

### Supernatant effects are pronounced under iron-limited conditions

**Figure 2:**
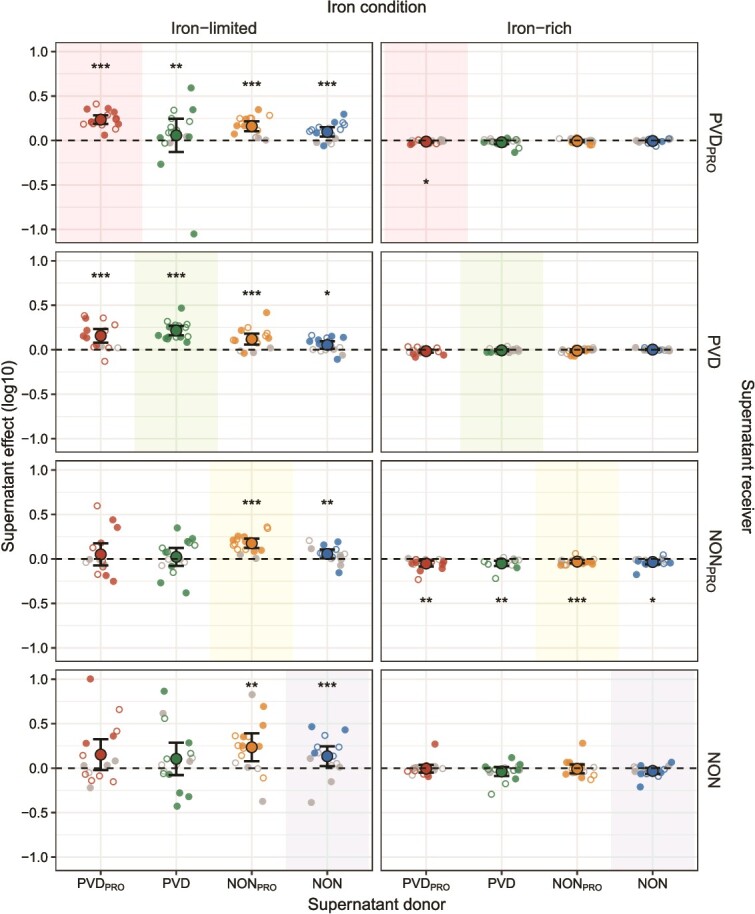
**Secreted compounds have pronounced effects under iron-limitation.** Shown are effects that PVD_PRO_, PVD, NON_PRO_, and NON strains isolated from soil (empty small circles) and freshwater (filled small circles) communities have on each other’s growth through compounds secreted into the supernatant under iron-limited and iron-rich conditions. Shaded rectangles highlight the effects that supernatants have on the growth of their own producer. Small circles show the median of four replicates obtained for each donor/receiver combination. Small grey circles show supernatant effects of specific donor/receiver combinations that did not differ from neutrality, whereas small colored circles indicate significant effects on receiver growth. Large circles and black lines show mean and confidence interval, respectively. Dashed horizontal lines indicate the null line where compounds in the supernatant have no effect on receiver growth. Asterisks above and below the null line indicate that the average supernatant effect of a specific combination of donor and receiver types was significantly positive and negative, respectively (significance levels are indicated as follows: ^*^0.05 ≥ *P* > 0.01; ^**^0.01 ≥ *P* > 0.001; ^***^P ≤ 0.001; see Table S7, S8 for further details).

**Figure 3:**
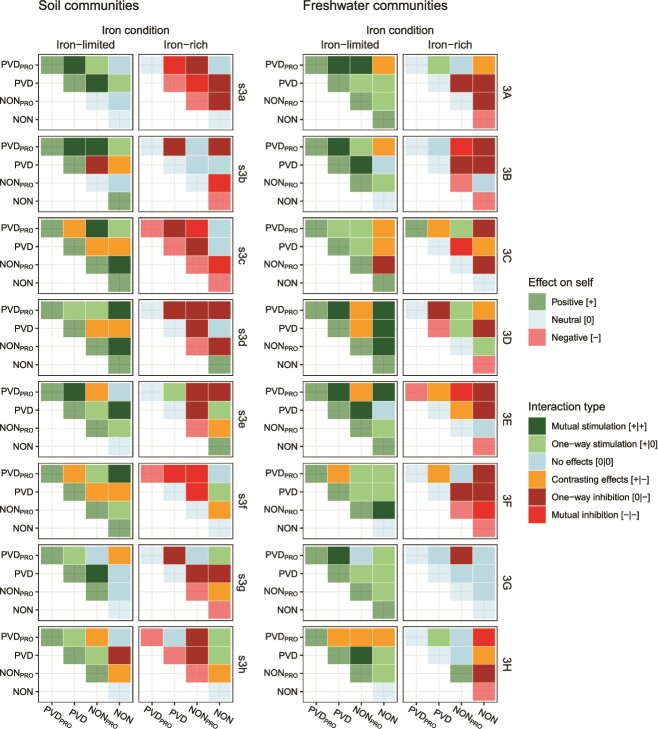
**Interaction types illustrate the high potential for growth stimulation under iron limitation.** Interaction types between – and effects on self of – PVD_PRO_, PVD, NON_PRO_, and NON strains from eight soil (s3a to s3h) and eight freshwater (3A to 3H) communities of *Pseudomonas* bacteria. Interaction types (opaque colors) and effects on self (transparent colors) were assigned based on the positive, neutral, or negative effects that the strains had on each other and themselves through compounds secreted into the supernatant under iron-limited and iron-rich conditions (see Fig. 2 and the methods for details).

We harvested supernatants from each strain and measured their effects on the growth of all community members (Table S7, S8, Fig. 2). We first focus on the effects of supernatants on their own producers. Under iron-limited conditions, effects on self were pronounced and overall positive for all types, indicating that strains typically secrete pyoverdine or other compounds that favor their own growth. This was different under iron-rich conditions, where effects on self were typically neutral or negative and generally small (Table S8, Fig. 2). Focusing next on the impact of supernatants on other community members, we likewise found that effects under iron-rich conditions were neutral or negative and typically small, suggesting a low baseline production of toxic compounds. Under iron-limited conditions, however, supernatant effects on others varied substantially, ranging from strong inhibition to strong stimulation for specific strain combinations (Table S8, Fig. 2). To further explore differences between iron conditions, we classified all pairwise interactions within a community from mutual inhibition to mutual stimulation and displayed them as interaction heatmaps for each community and condition (Fig. 3). An in-depth analysis revealed that many more positive strain interactions occur under iron-limited than iron-rich conditions, and that pyoverdine producers (PVD_PRO_ and PVD) more often benefit from supernatants than pyoverdine non-producers (NON_PRO_ and NON) across iron conditions (Fig. S5).

### Identity effects explain more variation in community functioning than strain interactions

We examined the effects of strain identity and strain interactions on two metrics of community functioning, productivity and pyoverdine production. To this end, we set up monocultures and cultures of all possible combinations of two, three, and four strains per community, and then used an established linear model method [24] to determine the variance explained by strain identity and strain interactions. We considered three measures of strain interactions: (i) non-linear strain richness, a statistical proxy for interactions [24]; (ii) the interaction score, a proxy reflecting the overall sign of supernatant effects within communities (Fig. 3); and (iii) a combination of the mean absolute supernatant effect and the proportion of positive effects, which accounts for both strength and sign of strain interactions.

**Figure 4:**
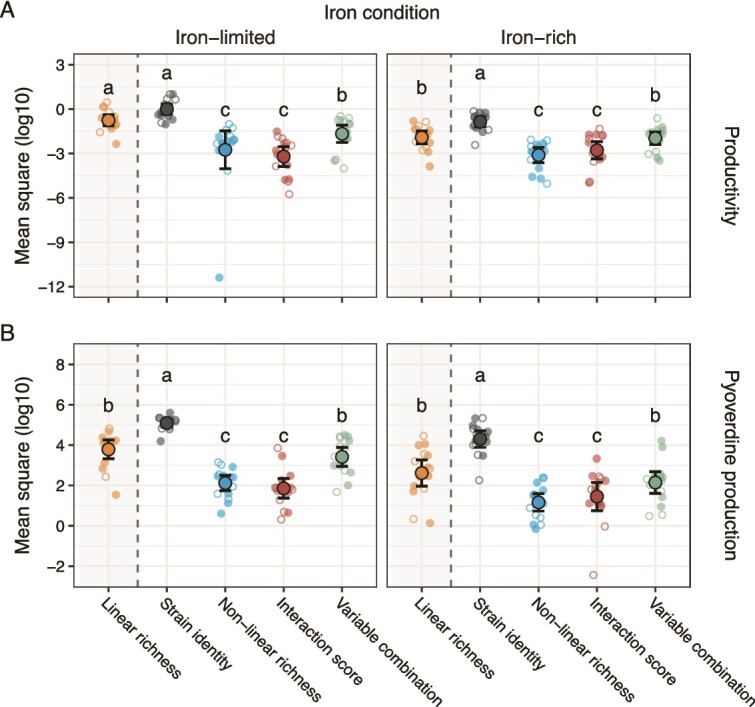
**Strain identity explains more variation in community productivity than strain interactions.** Shown are mean square values extracted from linear models fit for each of eight soil (empty small circles) and eight freshwater (filled small circles) communities to explain the impact of strain identity and three measures of strain interactions, non-linear richness, the interaction score, and a combination of variables on (A) community productivity and (B) pyoverdine production. The impact of linear richness, i.e. the extent to which community functioning linearly increases with strain richness, is shown for comparison (grey-shaded area). Whereas non-linear richness is a purely statistical proxy for strain interactions, the interaction score and the combination of variables are based on supernatant effects. The interaction score combines information on the sign and strength of supernatant effects, whereas the combination of variables includes the mean absolute supernatant effect and the proportion of positive supernatant effects and thus separately accounts for sign and strength. Large circles and black lines show means and confidence intervals. Small circles show mean square values obtained for specific communities from models fit separately to data generated under iron-limited and iron-rich conditions (letters show significantly different impacts on functioning based on the results of our statistical models; see the methods and Table 1, S9 for details).

We found that strain identity explained more variation in community productivity (Fig. 4A) and pyoverdine production (Fig. 4B) than strain interactions, regardless of which measure of strain interactions we considered, and across iron conditions (Table 1, S9). When comparing the three measures of strain interactions, we found that the combination of mean absolute supernatant effect and the proportion of positive supernatant effects explained more variation in functioning than both non-linear strain richness and the interaction score (Table 1, Fig. 4). Independent of these effects, strain identity explained more variation in productivity – and all predictors explained more variation in pyoverdine production – under iron-limited than iron-rich conditions (Table S9, Fig. 4).

**Table 1:** **Impact of strain identity and interactions on community functioning.** Post-hoc comparisons of different determinants of (A) productivity and (B) pyoverdine production of 16 small *Pseudomonas* communities. Significant *P*-values are in bold.

**(A) productivity**					
**contrast**	**condition**	**ratio**	**SE**	***t***_**135**_	***P***
strain ID - non-linear richness	iron-limited	563.78	489.16	7.301	**< 0.001**
strain ID - interaction score	iron-limited	1609.72	1396.67	8.510	**< 0.001**
strain ID - combined effects	iron-limited	46.29	40.16	4.420	**< 0.001**
non-linear richness - interaction score	iron-limited	2.86	2.48	1.209	0.229
non-linear richness - combined effects	iron-limited	0.08	0.07	−2.881	**0.007**
interaction score - combined effects	iron-limited	0.03	0.02	−4.090	**< 0.001**
strain ID - non-linear richness	iron-rich	178.87	155.20	5.978	**< 0.001**
strain ID - interaction score	iron-rich	83.95	72.84	5.106	**< 0.001**
strain ID - combined effects	iron-rich	12.97	11.25	2.953	**0.007**
non-linear richness - interaction score	iron-rich	0.47	0.41	−0.872	0.428
non-linear richness - combined effects	iron-rich	0.07	0.06	−3.024	**0.007**
interaction score - combined effects	iron-rich	0.15	0.13	−2.153	**0.041**
**(B) pyoverdine production**					
**contrast**		**ratio**	**SE**	***t***_**139**_	***P***
strain ID - non-linear richness		1139.78	569.86	14.078	**< 0.001**
strain ID - interaction score		1104.74	552.34	14.015	**< 0.001**
strain ID - combined effects		82.22	41.11	8.819	**< 0.001**
non-linear richness - interaction score		0.97	0.48	−0.062	0.950
non-linear richness - combined effects		0.07	0.04	−5.259	**< 0.001**
interaction score - combined effects		0.07	0.04	−5.196	**< 0.001**

### Strain types vary in their impact on community productivity

Thus far, we have shown that strain identity effects outweigh strain interaction effects on community functioning. However, this result does not reveal whether strain types differ in their impact on functioning across communities. To tackle this question, we again leveraged the above-described linear model method, this time focusing on the strain ID coefficients extracted from models using either community productivity, pyoverdine production, or the deviations from their expected values as functions of interest. The strain ID coefficients provide a measure of each strain’s effect on community functioning relative to an average strain’s contribution [24].

**Figure 5:**
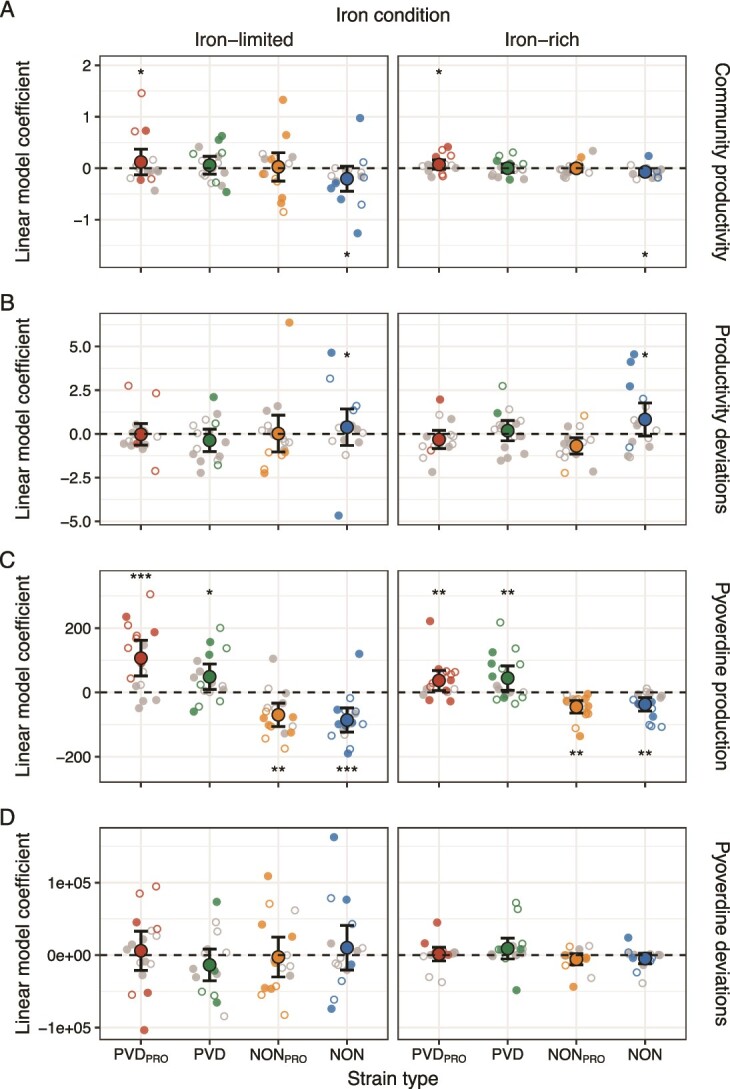
**Strain types and individual strains differ in their impact on community productivity.** Shown are linear model coefficients indicating the effect that PVD_PRO_, PVD, NON_PRO_, and NON strains from soil (empty small circles) and freshwater (filled small circles) communities had, relative to the average in their community, on (A) community productivity, (B) deviations from expected community productivity, (C) pyoverdine production, and (D) deviations from expected pyoverdine production. Large circles and black lines show means and confidence intervals. Dashed lines indicate average strain effects. Small circles show linear model coefficients obtained for specific strains from models fit separately to data generated for each community under iron-limited and iron-rich conditions, respectively. Small grey circles indicate that linear model coefficients were not significant, whereas small colored circles indicate significant coefficients. Asterisks above and below the average effect line indicate that the average effect of a specific type was significantly positive and negative, respectively (significance levels are based on the results of our statistical models and indicated as follows: ^*^0.05 ≥ *P* > 0.01; ^**^0.01 ≥ *P* > 0.001; ^***^P ≤ 0.001; statistics for specific strains are given in Table S11).

We found that PVD_PRO_ made above-average contributions to community productivity across iron conditions (main effect of type: *χ*^2^_3_ = 10.94, *P* = 0.012; PVD_PRO_: *t*_72.8_ = 2.22, *P* = 0.029; Fig. 5A), whereas NON contributed less than average (*t*_72.8_ = −2.44, *P* = 0.017; Fig. 5A). The presence of NON strains was generally associated with higher-than-average deviations from expected productivity (main effect of type: *χ*^2^_3_ = 7.89, *P* = 0.048; NON: *t*_124_ = 2.36, *P* = 0.020; Fig. 5B). When focusing on pyoverdine production, we observed that pyoverdine producers and non-producers made, respectively, above-average and below-average contributions across iron conditions (main effect of type: *χ*^2^_3_ = 87.64, *P* < 0.001; type-condition interaction: *χ*^2^_3_ = 13.39, *P* = 0.004; Table S10; Fig. 5C). In addition to these strain type effects, we observed that individual strains of each strain type variously featured above-average or below-average contributions to all measures of functioning (Table S11; Fig. 5).

### Interaction scores can predict productivity across communities

**Figure 6:**
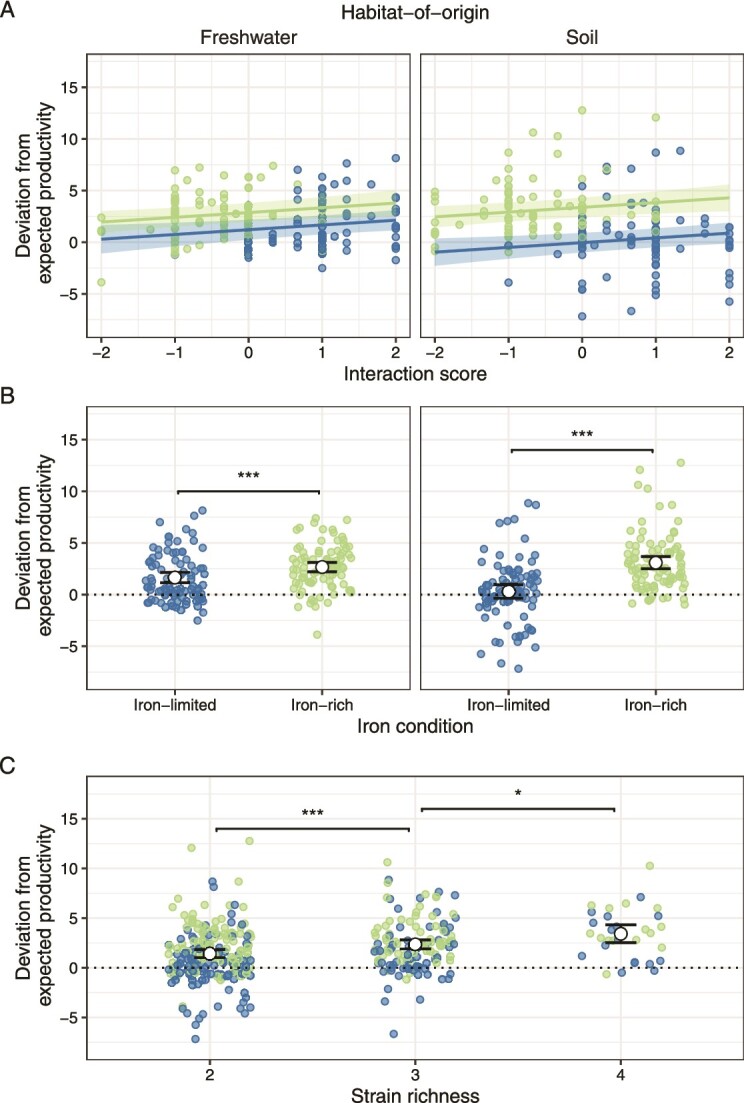
**Deviations from expected productivity increase as supernatant-based interactions shift from inhibitory to stimulatory.** Shown are relationships between deviations from expected community productivity and (A) the supernatant-based interaction scores, (B) iron condition, and (C) strain richness, as measured under iron-limited (blue) and iron-rich (green) conditions in freshwater and soil strains [left and right panels in (A) and (B), respectively]. High interaction scores indicate that stimulatory effects of secreted compounds prevail, whereas low interaction scores indicate a prevalence of inhibitory effects. Solid lines and shaded areas in (A) are regression lines and 95% confidence intervals, respectively. Large white circles and black lines in (B) and (C) show means and confidence intervals, respectively. Significance levels in (B) and (C) are indicated as follows: ^*^0.05 ≥ *P* > 0.01; ^**^0.01 ≥ *P* > 0.001; ^***^P ≤ 0.001.

Our findings show that strain interactions have a smaller impact on functioning than strain identity, but this does not mean that strain interactions are insignificant. In a last step, we therefore tested whether strain interactions had a consistent impact on functioning across communities. We focused on deviations from expected productivity (based on co-culture and mono-culture growth) and used the interaction score (based on supernatant effects) as a measure of interactions among community members. We predicted that low (inhibitory) and high (stimulatory) interaction scores should be associated with reduced and increased productivity deviations, respectively. Indeed, we found a positive relationship between deviations from expected productivity and the interaction score (Table S12; slope ± SE: 0.46 ± 0.18, *t*_331_ = 2.59, *P* = 0.010, Fig. 6A). Independent of this effect, productivity deviations were higher under iron-rich than under iron-limited conditions (Table S12; soil: *t*_331_ = 8.13, *P* < 0.001; freshwater: *t*_331_ = 3.91, *P* < 0.001; Fig. 6B) and increased with strain richness (Table S12; two vs. three strains: *t*_331_ = 3.67, *P* < 0.001; three vs. four strains: *t*_331_ = 2.36, *P* = 0.019; Fig. 6C).

## Discussion

Although microbial communities shape critical ecosystem services such as primary production and the fixation of greenhouse gases, we are only beginning to understand how these communities function. Here, we compared the impact of strain interactions and strain identity on the functioning of 16 *Pseudomonas* communities, each comprising four strains of distinct types varying in their potential to secret the siderophore pyoverdine and proteases. We found that strain identity effects explained more variation in community productivity and pyoverdine production than strain interactions, regardless of whether interactions were inferred statistically or measured more directly using supernatant assays. Although different strain types overall affected functioning in accordance with their baseline (monoculture) growth and pyoverdine production, we also found that individual strains deviated from this pattern in co-culture, suggesting that strain interactions can modulate the impact of strain identity. Indeed, we found that deviations from expected productivity consistently increased across communities as interactions through secreted compounds shifted from inhibitory to stimulatory. Altogether, our findings suggest that although strain identity effects may often outweigh effects of strain interactions, both factors will usually be required to gain a nuanced understanding of how diverse natural communities function.

Classical studies of sociomicrobiology focusing on closely related strains have consistently reported that strain interaction effects can outweigh the impact of strain identity on functioning (e.g., [48–51]). For instance, Jiricny et al. [48] showed that a pyoverdine-producing *P. aeruginosa* strain grew to higher density than an isogenic non-producer in monoculture, but was outcompeted by that non-producer in mixed culture because the non-producer could exploit the pyoverdines secreted by producers. This strong strain interaction effect was observed because the interacting strains were identical (apart from pyoverdine production), thereby ruling out any strain identity effects that are unlinked to the social trait. By contrast, in our study we included genetically more diverse *Pseudomonas* strains and found that social interactions have comparably small effects on community functioning compared the pronounced effects of strain identity. Identity effects often result from differences in individual-level traits including growth rate and metabolic capabilities [52]. Such differences are expected to increase with strain genetic divergence [53, 54]. Our findings thus suggest that the impact of strain interactions relative to strain identity declines when moving from genetically homogenous to more diverse communities characterized by lower relatedness.

We have previously shown that the siderophores produced by *Pseudomonas* isolates mediate diverse and strong strain interactions [8]. Our supernatant assays indeed revealed greater scope for such interactions under iron-limited than iron-rich conditions. We therefore anticipated strain interactions to have a stronger impact on community functioning under iron limitation. However, we found little evidence that iron availability affected the relative impact of social interactions on functioning. A potential explanation for this mismatch could be social trait linkage. We have previously shown that the production of different secreted compounds, including pyoverdines, proteases, biofilm, and toxic compounds, are often positively correlated among our natural isolates [28]. Consequently, strains contributing a lot to pyoverdine production under iron limitation may also contribute a lot to other secreted compounds under iron rich conditions, leading to consistent (large or small) effects of specific strains on functioning across conditions. In support of this idea, we found that PVD_PRO_ strains contributed more – and NON strains less – to productivity than the average strain regardless of iron condition. Hence, positive trait linkage may stabilize the impact of individual community members on functioning across environmental conditions.

We found that deviations from expected productivity increased when supernatant effects shifted from inhibition to stimulation, indicating that mutually stimulatory effects of secreted compounds could promote community functioning. One possible explanation is that such mutual stimulation entails mutual benefits, for example through the increased availability of growth-limiting resources for strains with non-overlapping niche requirements [54, 55]. However, mutual stimulation can also arise because of reciprocal exploitation, whereby each strain benefits from a publicly available compound secreted by the other strain without paying the associated production costs [56]. Such mutual exploitation [57] could promote higher than expected functioning at the community level if one strain derives net benefits from the interaction that outweigh the net costs to the other strain(s) [58, 59]. Overall, these considerations highlight that the secretion of sharable” public goods” might often strongly affect the functioning of bacterial communities even when identity effects are strong.

To quantify strain interactions effects on community functioning, we used non-linear strain richness as a statistical proxy for interactions [24] and compared its impact with experimentally inferred interaction measures based on supernatant effects. We found that non-linear richness performed as well as our univariate interaction score, but captured less variation in functioning than a combination of two variables separately capturing both the strength and sign of supernatant effects. These findings validate both non-linear richness and the interaction score as simple measures of strain interactions. However, they also show that separately accounting for the strength and sign of interactions can provide additional benefits in estimating the impact of strain interactions on community functioning. One plausible reason is that estimates of non-linear richness and the interaction score sometimes cannot differentiate between distinct interaction patterns resulting in the same overall outcome. For instance, pairwise [0/0] and [+/−] interactions would both yield an interaction score of zero. We therefore recommend obtaining experimental data (strength and sign) when the impact of strain interactions is of particular interest, but to rely on non-linear richness as proxy when it is not.

In recent years, interest in manipulating microbial assemblies and microbiomes to perform beneficial functions has skyrocketed [60–63] and the use of probiotics has become popular in agriculture [64], aquaculture [65] and human health [66]. Proposed approaches often make use of microbial interactions with the idea to introduce or promote probiotic strains in communities that engage in competition with pathogens [35] or exploit their social traits [67]. Such interactions are often driven by secreted secondary metabolites such as toxins (interference competition; [35]) and siderophores (exploitation; [68]). Although conceptually appealing, our results highlight that cheating mutants might only be successful if they possess additional individual-level traits that allow them to be competitive in their niche regardless of their exploitative abilities. More generally, our findings suggest that strain identity effects, in addition to social interactions, must be considered when developing powerful and sustainable probiotics.

In conclusion, we showed that strain identity effects have a larger impact on the productivity and pyoverdine production of *Pseudomonas* communities than strain interactions. Our results suggest that strain interactions might often reinforce or diminish, but rarely overwrite, existing baseline differences in how diverse microbial players affect the functioning of their community. Although our study solely focused on *Pseudomonas* bacteria, we anticipate even stronger identity effects on functioning in taxonomically more diverse microbial communities. At the same time, relative impact patterns may vary across functions. Whereas productivity often resembles a zero-sum game – one strain can only increase its contribution to functioning at the expense of another – other functions may leave more scope for net-positive gains (e.g., community respiration; [24]). Such functions should be determined to a greater extent by species interactions relative to identity effects. Future work should therefore consider multiple functions in addition to productivity to unravel the importance of species interactions in natural microbial communities of varying taxonomical diversity.

## Supplementary Material

Kramer_FunctioningDeterminants_Supplements_Final_wraf025

TableS1_PhylogeneticDistance_wraf025

TableS2_SupernatantEffectNature_wraf025

TableS3_FinalModelStructure_wraf025

TableS11_StrainContributions_wraf025

## Data Availability

The data supporting the findings of this study are available in the Dryad repository (http://doi.org/10.5061/dryad.1c59zw44p).
