# Supplementary material for: Strain identity effects contribute more to Pseudomonas community functioning than strain interactions
Source: ISME J. 2025 Feb 8;19(1):wraf025. doi: 10.1093/ismejo/wraf025 (PMC11879211; doi:10.1093/ismejo/wraf025)
Supplement: Kramer_FunctioningDeterminants_Supplements_Final_wraf025 [file kramer_functioningdeterminants_supplements_final_wraf025.pdf]

Supplementary Material

Strain identity effects contribute more to *Pseudomonas* community functioning than strain interactions

Jos Kramer[1,2,§], Simon Maréchal[1] , Alexandre R.T. Figueiredo[1,3] and Rolf Kümmerli[1]

[1] Department of Quantitative Biomedicine, University of Zurich, Switzerland

[2] Department of Environmental Systems Science, ETH Zurich, Switzerland

[3] Department of Biology, University of Oxford, United Kingdom

[§] corresponding author: Jos Kramer, email: JosKramer@gmx.de; phone: +41446328922

This file contains the following Supplementary Information:

- **Supplementary Methods**
- **Supplementary Analyses**
- **Supplementary References**
- **Supplementary Table S4-S10 + Table S12**

  [Tables S1, S2, S3 and S11 are available as separate files]
- **Supplementary Figures S1-S5**

Supplementary Methods

Strain selection. We selected strains from an established collection of 315 *Pseudomonas* strains isolated from eight soil and eight freshwater samples (18-20 strains per sample; hereafter: community) [1, 2]. From each of these 16 communities, we selected four strains based on their production of pyoverdine and proteases. Per community, we chose the most divergent strains within the observed phenotype space, aiming to select (i) one strain producing pyoverdine and proteases [PVD_{PRO}], (ii) one strain producing only pyoverdine [PVD], (iii) one strain producing only proteases [NON_{PRO}], and (iv) one strain producing neither pyoverdine nor proteases [NON]. To quantify pyoverdine production, we grew all 315 strains in iron-limited casamino acids (CAA) medium and measured the natural fluorescence of pyoverdine in the culture supernatant. To quantify protease production, we spotted aliquots of bacterial culture onto skim milk agar and then measured the size of the proteolytic halo forming around protease-producing colonies (see [3] for details). Based on the pyoverdine and protease production values, we assigned one of the four types to each strain, and then picked one strain per type for each community. When the available types were not clearly defined (e.g., because no complete non-producers of pyoverdine and proteases where present in the focal community), we picked a strain with a profile close to the intended type. When possible, we included previously sequenced strains [1]. Overall, the pyoverdine and protease production of selected strains matched the intended type, with PVD_{PRO} and PVD typically producing pyoverdine at higher levels than NON_{PRO} and NON (Figure S2).

Strain phylogeny. To verify that we selected a diverse set of strains, we quantified the phylogenetic distance between strains of the same habitat (soil or freshwater) based on partial sequences of the *rpoD* gene. We first obtained the partial *rpoD* gene sequences of the selected strains from the European Nucleotide Archive (generated by [1]; Accession number: PRJEB21289). Next, we used the DistanceMatrix function (DECIPHER package; [4]) to calculate the pairwise dissimilarity between, respectively, all freshwater and all soil isolates, and then transformed the default dissimilarity measures into similarity measures using a "1-Matrix" operation. To assess the diversity of strains within our experimental communities, we checked whether strains from the same community belonged to the

same or different species, using a pairwise similarity threshold of 96% to identify conspecifics [5, 6]. We found that strains within communities generally represent different species, with only one exception (in soil community s3d, PVD and NON_{PRO} showed a pairwise similarity of 97% despite marked differences in their phenotype; Table S1, Figure S1+S2).

To display the phylogenetic diversity of our *Pseudomonas* strains, we generated phylogenetic trees for soil and freshwater strains (Figure S1). First, we obtained *rpoD* gene sequences of five reference *Pseudomonas* strains from the Pseudomonas.com database [7] . We then used the subseq function (XVector package; [8]) to trim the full-length *rpoD* sequences of these type-strains at positions 488 and 1044, so that their length would match the partial sequences of our natural isolates. Next, we generated two alignment objects (for freshwater and soil strains, each with the type strains) using the AlignSeqs function (DECIPHER package), and then ran the alignments through the phyDat function (phangorn package; [9]) to transform them into phyDat objects. Finally, we ran the phyDat objects through the modelTest function and used the output to generate Maximum Likelihood trees with the pml_bb function, subsequently bootstraped with the bootstrap.pml function (phangorn package). After rooting the trees at their midpoints, we generated the tree figures with the ggtree package [10]. To quantify the extent to which phylogeny predicts pyoverdine and protease production profiles, we finally calculated and compared Abouheif's C_{mean} for each trait and habitat (abouheif.moran function, adephylo package; [11]). C_{mean} values close to one reflect strong phylogenetic signals, suggesting that phylogenetic history is the main factor driving trait covariance among strains. Conversely, C_{mean} values close to zero reflect weak signals, suggesting that factors apart from phylogeny affect trait expression.

Quantification of total siderophore production. To quantify the total iron-chelating activity of our strains, we used the Chrome Azurol Sulfonate (CAS) assay [12]. This colorimetric assay uses an Fe(III)-Chrome Azurol Sulfonate-Hexadecyltrimethylammonium bromide [Fe(III)-CAS-HDTMA] complex that changes color, from blue to orange, when it is deferrated by exchange reactions with iron-chelating compounds such as siderophores. We first collected supernatant generated by each strain under iron-

limited and iron-rich conditions (details in the main text), and then transferred 20 µL of each supernatant into 240 µL of CAS assay solution (prepared according to the original recipe; see [12]). We incubated these mixtures at room temperature in the dark for 30 minutes, and then quantified the total iron-chelating activity, a proxy measure of the total production of siderophores, by measuring the loss of the blue-colored Fe-CAS-HDTMA complex at 630 nm.

Supplementary Analyses

Total siderophore production of strain types. In the main text, we show that pyoverdine production differs between producer (PVD_{PRO} and PVD) and non-producer types (NON_{PRO} and NON). However, pseudomonads sometimes deploy additional siderophores to scavenge for iron. To account for this possibility, we measured the total iron-chelating activity of each strain as a proxy for the production of all siderophores (see above), and then examined whether it differed between types, experimental conditions or habitat-of-origin. We found that all these factors jointly affected siderophore production (habitat: $\chi^2_1 = 0.63$, $P = 0.427$; condition: $\chi^2_1 = 348.18$, $P < 0.001$; donor type: $\chi^2_3 = 0.06$, $P = 0.996$; interaction habitat x donor type: $\chi^2_3 = 8.72$, $P = 0.033$; interaction condition x donor type: $\chi^2_3 = 49.25$, $P < 0.001$). Under iron-limited conditions, PVD_{PRO} and NON featured, respectively, the highest and lowest siderophore production, whereas PVD and NON_{PRO} produced siderophores at an intermediate level (Table S5; Figure S4). This latter result seems at odds with the growth differences between PVD and NON_{PRO} reported in the main text. However, the CAS assay measures any form of iron-chelation activity, also by compounds that chelate iron but do not deliver it back to cells. The CAS values are therefore not expected to strongly correlate with growth benefits. Moreover, secondary siderophores (e.g., pyochelin and yersiniabactin) produced by pyoverdine non-producers are typically less potent for iron acquisition than pyoverdine and thus need to be produced in higher amounts to achieve the same iron-chelating activity [13]. In contrast to the situation under iron-limited conditions, siderophore production under iron-rich conditions did not differ between types and was generally low (Table S5; Figure S4). Although differences between strains from soil versus freshwater habitats were generally

small (Figure S4), we moreover found that PVD strains from freshwater featured lower pyoverdine production than PVD strains from soil ($t_{48.1}$= -2.877, P = 0.006).

Phylogenetic signal in pyoverdine and protease production. We calculated Abouheif's C_{mean} to test to what extent phylogeny predicted variation in pyoverdine and protease production. We found that neither pyoverdine (freshwater: C_{mean} = -0.193, P = 0.957; soil: C_{mean} = 0.011, P = 0.437) nor protease production (freshwater: C_{mean} = 0.029, P = 0.388; soil: C_{mean} = -0.169, P = 0.953) feature a significant phylogenetic signal, indicating that trait expression may frequently change within clades (Figure S1). Although this result seems at odds with our previous study reporting intermediate phylogenetic signals for both traits [3], it could simply reflect the lower sample size in the present study (64 vs. 315 strains). Alternatively (or additionally), our sampling of phenotypically and phylogenetically diverse strains could have removed any phylogenetic bias in the strain subset we used here.

Relative frequencies of supernatant-based interaction types. We used ordered logistic regression (vglm function, VGAM package; [14]) to analyze whether the proportions of stimulatory, neutral, and inhibitory interactions depended on media conditions (iron-limited or iron-rich) and whether the supernatant donors and receivers are pyoverdine producers (PVD_{PRO} and PVD) or non-producers (NON_{PRO} and NON). We initially fitted community as an additional main effect (without interactions) to account for the non-independence of strains from the same community, but dropped it during model simplification because it was not significant (χ^2_{30} = 34.06, P = 0.279). We found that supernatant effects were generally higher under iron-limited rather than iron-rich conditions (estimate ± SE; negative vs. neutral: 1.483 ± 0.264, z = 5.619, P < 0.001; neutral vs. positive: 2.307 ± 0.270, z = 8.534, P < 0.001) and when the supernatant receiver was a pyoverdine producer rather than non-producer (negative vs. neutral: 0.837 ± 0.266, z = 3.146, P = 0.002; neutral vs. positive: 0.937 ± 0.260, z = 3.610, P < 0.001; Figure S5). Conversely, supernatant effects were somewhat lower when the supernatant donor was a pyoverdine producer (negative vs. neutral: -0.744 ± 0.266, z = -2.794, P = 0.005; neutral vs. positive:

0.038 ± 0.256, z = 0.148, P = 0.882; Figure S5). Together, these results suggest that the effects of secreted components are mostly determined by the receiver's ability to make use of them.

Supplementary References

1. Butaitė E, Baumgartner M, Wyder S, Kümmerli R. Siderophore cheating and cheating resistance shape competition for iron in soil and freshwater *Pseudomonas* communities. *Nature Communications* 2017; **8**: 414.
2. Butaitė E, Kramer J, Wyder S, Kümmerli R. Environmental determinants of pyoverdine production, exploitation and competition in natural *Pseudomonas* communities. *Environ Microbiol* 2018; **20**: 3629–3642.
3. Kramer J, López Carrasco MÁ, Kümmerli R. Positive linkage between bacterial social traits reveals that homogeneous rather than specialised behavioral repertoires prevail in natural *Pseudomonas* communities. *FEMS Microbiology Ecology* 2020; **96**: fiz185.
4. Wright ES. Using DECIPHER v2.0 to analyze big biological sequence data in R. *The R Journal* 2016; **8**: 352.
5. Mulet M, Gomila M, Ramírez A, Cardew S, Moore ERB, Lalucat J, et al. Uncommonly isolated clinical *Pseudomonas*: identification and phylogenetic assignation. *Eur J Clin Microbiol Infect Dis* 2017; **36**: 351–359.
6. Sánchez D, Matthijs S, Gomila M, Tricot C, Mulet M, García-Valdés E, et al. *rpoD* gene pyrosequencing for the assessment of *Pseudomonas* diversity in a water sample from the Woluwe river. *Appl Environ Microbiol* 2014; **80**: 4738–4744.
7. Winsor GL, Griffiths EJ, Lo R, Dhillon BK, Shay JA, Brinkman FSL. Enhanced annotations and features for comparing thousands of *Pseudomonas* genomes in the *Pseudomonas* genome database. *Nucleic Acids Res* 2016; **44**: D646–D653.
8. Pagès H, Aboyoun P. XVector: Foundation of external vector representation and manipulation in Bioconductor. 2024. R package version 0.46.0. https://bioconductor.org/packages/XVector.
9. Schliep KP. phangorn: phylogenetic analysis in R. *Bioinformatics* 2011; **27**: 592–593.
10. Yu G, Smith DK, Zhu H, Guan Y, Lam TT. GGTREE : an R package for visualization and annotation of phylogenetic trees with their covariates and other associated data. *Methods Ecol Evol* 2017; **8**: 28–36.
11. Jombart T, Balloux F, Dray S. *adephylo* : new tools for investigating the phylogenetic signal in biological traits. *Bioinformatics* 2010; **26**: 1907–1909.
12. Schwyn B, Neilands JB. Universal chemical assay for the detection and determination of siderophores. *Analytical Biochemistry* 1987; **160**: 47–56.

13. Kümmerli R. Iron acquisition strategies in pseudomonads: mechanisms, ecology, and evolution. *Biometals* 2023; **36**: 777–797.
14. Yee T. VGAM: Vector Generalized Linear and Additive Models. 2024. R package version 1.1-12, https://CRAN.R-project.org/package=VGAM.

Table S4 | Growth and siderophore production profiles. Determinants of (A) monoculture growth and (B) pyoverdine production under iron-rich and iron-limited conditions of soil and freshwater *Pseudomonas* strains belonging to four different strain types (PVD_{PRO}, PVD, NON_{PRO}, and NON) varying in their production of proteases and the siderophore pyoverdine. Significant *P*-values are in bold.

	**(A) growth**			**(B) pyoverdine production**		
	**df**	χ^2	***P***	**df**	χ^2	***P***
**habitat**	1	5.28	**0.022**	1	3.01	0.083
**condition**	1	217.93	**< 0.001**	1	267.68	**< 0.001**
**strain type**	3	65.90	**< 0.001**	3	115.62	**< 0.001**
**habitat : strain type**	3	9.75	**0.021**	-	-	-
**condition : strain type**	3	9.79	**0.021**	3	122.89	**< 0.001**

Table S5 | Growth and pyoverdine production of strain types. Post-hoc comparisons of differences in (A) growth and (B) pyoverdine production between four strain types (PVD_{PRO}, PVD, NON_{PRO}, and NON) and iron conditions. *P*-values are adjusted for multiple testing using the false discovery rate. Significant *P*-values are in bold print. Note that, independent of the comparisons below, NON strains from freshwater grew, on average, better than NON strains from soil (0.09 ± 0.04, $t_{91.1}$ = 2.252, *P* =0.027).

(A) growth

contrast	condition	estimate	SE	df	t	*P*
PVD_{PRO} - PVD	iron-limited	0.09	0.03	56.23	2.557	**0.016**
PVD_{PRO} - NON_{PRO}	iron-limited	0.17	0.03	56.23	4.989	**< 0.001**
PVD_{PRO} - NON	iron-limited	0.27	0.03	56.23	8.017	**< 0.001**
PVD - NON_{PRO}	iron-limited	0.08	0.03	56.23	2.432	**0.018**
PVD - NON	iron-limited	0.18	0.03	56.23	5.460	**< 0.001**
NON_{PRO} - NON	iron-limited	0.10	0.03	56.23	3.028	**0.006**
PVD_{PRO} - PVD	iron-rich	0.04	0.06	58.22	0.637	0.632
PVD_{PRO} - NON_{PRO}	iron-rich	-0.01	0.06	58.22	-0.179	0.859
PVD_{PRO} - NON	iron-rich	0.11	0.06	58.22	1.914	0.182
PVD - NON_{PRO}	iron-rich	-0.05	0.06	58.22	-0.816	0.627
PVD - NON	iron-rich	0.07	0.06	58.22	1.277	0.413
NON_{PRO} - NON	iron-rich	0.12	0.06	58.22	2.093	0.182

contrast	donor type	estimate	SE	df	t	*P*
iron-limited vs. iron-rich	PVD_{PRO}	-0.25	0.05	93.71	-5.352	**< 0.001**
iron-limited vs. iron-rich	PVD	-0.30	0.05	93.71	-6.383	**< 0.001**
iron-limited vs. iron-rich	NON_{PRO}	-0.43	0.05	93.71	-9.104	**< 0.001**
iron-limited vs. iron-rich	NON	-0.41	0.05	93.71	-8.687	**< 0.001**

(B) pyoverdine production

contrast	condition	estimate	SE	df	t	*P*
PVD_{PRO} - PVD	iron-limited	7.32	6.38	59.73	1.147	0.2558
PVD_{PRO} - NON_{PRO}	iron-limited	56.75	6.38	59.73	8.889	**< 0.001**
PVD_{PRO} - NON	iron-limited	71.51	6.38	59.73	11.2	**< 0.001**
PVD - NON_{PRO}	iron-limited	49.42	6.38	59.73	7.742	**< 0.001**
PVD - NON	iron-limited	64.19	6.38	59.73	10.05	**< 0.001**
NON_{PRO} - NON	iron-limited	14.76	6.38	59.73	2.313	**0.029**
PVD_{PRO} - PVD	iron-rich	1.60	1.88	58.89	0.85	0.4783
PVD_{PRO} - NON_{PRO}	iron-rich	10.40	1.88	58.89	5.54	**< 0.001**
PVD_{PRO} - NON	iron-rich	10.28	1.88	58.89	5.476	**< 0.001**
PVD - NON_{PRO}	iron-rich	8.80	1.88	58.89	4.69	**< 0.001**

PVD - NON	iron-rich	8.68	1.88	58.89	4.626	**< 0.001**
NON_{PRO} - NON	iron-rich	-0.12	1.88	58.89	-0.06	0.9494

**contrast**	**donor type**	**estimate**	**SE**	**df**	**t**	***P***
iron-limited vs. iron-rich	PVD_{PRO}	66.81	4.70	70.02	14.201	**< 0.001**
iron-limited vs. iron-rich	PVD	61.09	4.70	70.02	12.983	**< 0.001**
iron-limited vs. iron-rich	NON_{PRO}	20.47	4.70	70.02	4.351	**< 0.001**
iron-limited vs. iron-rich	NON	5.58	4.70	70.02	1.187	0.239

Table S6 | Total iron-chelating activity of strain types. Post-hoc comparisons of differences in total iron-chelating activity between four strain types (PVD_{PRO}, PVD, NON_{PRO}, and NON). *P*-values are adjusted for multiple testing using the false discovery rate. Significant *P*-values are in bold print.

contrast	condition	estimate	SE	df	t	*P*
PVD_{PRO} - PVD	iron-limited	0.11	0.05	59.05	2.456	**0.020**
PVD_{PRO} - NON_{PRO}	iron-limited	0.18	0.05	59.05	3.933	**< 0.001**
PVD_{PRO} - NON	iron-limited	0.31	0.05	59.05	6.596	**< 0.001**
PVD - NON_{PRO}	iron-limited	0.07	0.05	59.05	1.476	0.145
PVD - NON	iron-limited	0.19	0.05	59.05	4.140	**< 0.001**
NON_{PRO} - NON	iron-limited	0.12	0.05	59.05	2.663	**0.015**
PVD_{PRO} - PVD	iron-rich	0.01	0.01	45.81	0.545	0.588
PVD_{PRO} - NON_{PRO}	iron-rich	-0.01	0.01	45.81	-0.826	0.496
PVD_{PRO} - NON	iron-rich	-0.02	0.01	45.81	-1.668	0.307
PVD - NON_{PRO}	iron-rich	-0.02	0.01	45.81	-1.371	0.354
PVD - NON	iron-rich	-0.03	0.01	45.81	-2.213	0.192
NON_{PRO} - NON	iron-rich	-0.01	0.01	45.81	-0.842	0.496

Table S7 | Determinants of supernatant effects. Determinants of effects that donors had on receiver growth through compounds secreted into the supernatant under iron-limited and iron-rich conditions. Strains were isolated from soil or freshwater samples. Significant *P*-values are in bold print.

	χ^2	df	*P*
**condition**	419.21	1	**< 0.001**
**habitat**	3.69	1	0.055
**donor**	3.54	3	0.316
**receiver**	36.23	3	**< 0.001**
**condition : donor**	40.06	3	**< 0.001**
**condition : receiver**	6.29	3	0.098
**habitat : receiver**	8.57	3	**0.036**
**donor : receiver**	8.31	9	0.503
**condition : donor : receiver**	32.61	9	**< 0.001**

Table S8 | Supernatant effects of strain types. Post-hoc comparisons of differences in supernatant effects that strains of four types (PVD_{PRO}, PVD, NON_{PRO}, and NON) have on each other. *P*-values are adjusted for multiple testing using the false discovery rate. Significant *P*-values are in bold. Independent of the comparisons below, PRO strains from freshwater overall benefitted more from receiving supernatants from others than PRO strains from soil (ratio ± SE: 1.06 ± 0.03, t_{14} = 2.233, *P* = 0.043).

supernatant donor	supernatant receiver	condition	response	SE	t_{14}	*P*
PVD_{PRO}	PVD_{PRO}	iron-limited	1.72	0.09	9.784	**< 0.001**
PVD	PVD_{PRO}	iron-limited	1.38	0.13	3.341	**0.005**
NON_{PRO}	PVD_{PRO}	iron-limited	1.45	0.09	5.980	**< 0.001**
NON	PVD_{PRO}	iron-limited	1.27	0.07	4.326	**0.001**
PVD_{PRO}	PVD	iron-limited	1.46	0.11	5.152	**< 0.001**
PVD	PVD	iron-limited	1.65	0.08	10.043	**< 0.001**
NON_{PRO}	PVD	iron-limited	1.31	0.07	5.122	**< 0.001**
NON	PVD	iron-limited	1.13	0.05	2.706	**0.017**
PVD_{PRO}	NON_{PRO}	iron-limited	1.12	0.15	0.862	0.403
PVD	NON_{PRO}	iron-limited	1.05	0.09	0.601	0.557
NON_{PRO}	NON_{PRO}	iron-limited	1.50	0.08	7.392	**< 0.001**
NON	NON_{PRO}	iron-limited	1.15	0.05	3.275	**0.006**
PVD_{PRO}	NON	iron-limited	1.39	0.25	1.812	0.091
PVD	NON	iron-limited	1.34	0.19	2.046	0.060
NON_{PRO}	NON	iron-limited	1.58	0.20	3.646	**0.003**
NON	NON	iron-limited	1.49	0.13	4.490	**0.001**
PVD_{PRO}	PVD_{PRO}	iron-rich	0.98	0.01	-2.693	**0.017**
PVD	PVD_{PRO}	iron-rich	0.96	0.02	-2.040	0.061
NON_{PRO}	PVD_{PRO}	iron-rich	0.98	0.01	-1.473	0.163
NON	PVD_{PRO}	iron-rich	1.00	0.01	-0.143	0.889
PVD_{PRO}	PVD	iron-rich	0.98	0.01	-1.725	0.106
PVD	PVD	iron-rich	0.99	0.01	-1.303	0.214
NON_{PRO}	PVD	iron-rich	0.97	0.01	-1.769	0.099
NON	PVD	iron-rich	1.00	0.01	-0.038	0.970
PVD_{PRO}	NON_{PRO}	iron-rich	0.90	0.02	-3.929	**0.002**
PVD	NON_{PRO}	iron-rich	0.92	0.02	-3.978	**0.001**
NON_{PRO}	NON_{PRO}	iron-rich	0.93	0.01	-5.671	**< 0.001**
NON	NON_{PRO}	iron-rich	0.93	0.02	-3.449	**0.004**
PVD_{PRO}	NON	iron-rich	0.96	0.03	-1.391	0.186
PVD	NON	iron-rich	0.93	0.04	-1.613	0.129
NON_{PRO}	NON	iron-rich	0.97	0.04	-0.885	0.391
NON	NON	iron-rich	0.96	0.03	-1.184	0.256

Table S9 | Determinants of community functioning. Determinants of the variation explained in (A) community productivity and (B) pyoverdine production in series of linear models fit on data from each community (see the main text for details). *P*-values are adjusted for multiple testing using the false discovery rate. Significant *P*-values are in bold print. Note that strain identity explained more variation in productivity (t_{135} = 2.264, P = 0.025), and all predictors explained more variation in pyoverdine production (t_{139} = 6.700, P < 0.001), under iron-limited than iron-rich conditions.

	**(A) Productivity**			**(B) Pyoverdine production**		
	χ^2	**df**	***P***	χ^2	**df**	***P***
**habitat**	1.4	1	0.238	1.0	1	0.316
**DOI[§]**	133.8	4	**< 0.001**	272.3	4	**< 0.001**
**condition**	7.1	1	**0.008**	44.9	1	**< 0.001**
**name : condition**	10.4	4	**0.035**	-	-	-

[§]Determinant of interest; strain identity, non-linear richness, interaction score or variable combination

Table S10 | Contributions of different strain types to pyoverdine production. Results of linear models testing whether different strain types featured above- or below-average contributions to community pyoverdine production under iron-limited and iron-rich conditions. Significant *P*-values are in bold.

strain type	condition	emmean	SE	df	t	*P*
PVD_{PRO}	iron-limited	106.76	20.24	60.00	5.274	**< 0.001**
PVD	iron-limited	48.73	20.24	60.00	2.407	**0.019**
NON_{PRO}	iron-limited	-69.68	20.24	60.00	-3.442	**0.001**
NON	iron-limited	-85.81	20.24	60.00	-4.239	**< 0.001**
PVD_{PRO}	iron-rich	37.10	13.38	60.00	2.774	**0.007**
PVD	iron-rich	44.56	13.38	60.00	3.331	**0.001**
NON_{PRO}	iron-rich	-44.74	13.38	60.00	-3.345	**0.001**
NON	iron-rich	-36.92	13.38	60.00	-2.760	**0.008**

Table S12 | Deviations from expected community productivity. Determinants of deviations from expected community productivity. Significant *P*-values are in bold.

	χ^2_1	*P*
**habitat**	0.371	0.542
**condition**	54.792	**< 0.001**
**strain richness**	28.475	**< 0.001**
**interaction score**	6.725	**0.010**
**habitat : condition**	12.995	**< 0.001**

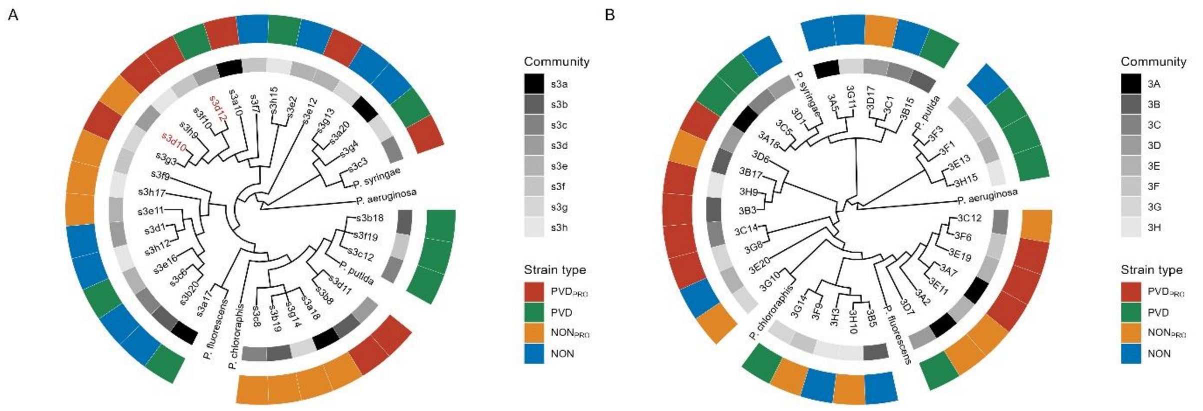

Figure S1 | Phylogenetic trees of natural *Pseudomonas* isolates. Maximum-likelihood cladograms for soil (A) and freshwater (B) strains based on partial *rpoD* sequences. For both habitats, published *rpoD* sequences of five well-characterised pseudomonads (*P. aeruginosa*, *P. syringae*, *P. putida*, *P. fluorescens*, and *P. chlororaphis*) were integrated into the cladograms to demonstrate taxonomic affiliation and the high diversity of our environmental isolates. The inner color strip indicates each strain's affiliation to a particular community [shades of grey]. The outer color strip indicates each strain's affiliation to one of four strain types (PVD_{PRO} [red], PVD [green], NON_{PRO} [orange], NON [blue]). Within communities, all strains belonged to distinct phylogenetic clades in all but one community (the two closely related strains in community s3d are printed in red). Note that neither pyoverdine production nor protease production underlying the classification of strains into strain types featured a significant phylogenetic signal (see the Supplementary Analyses above).

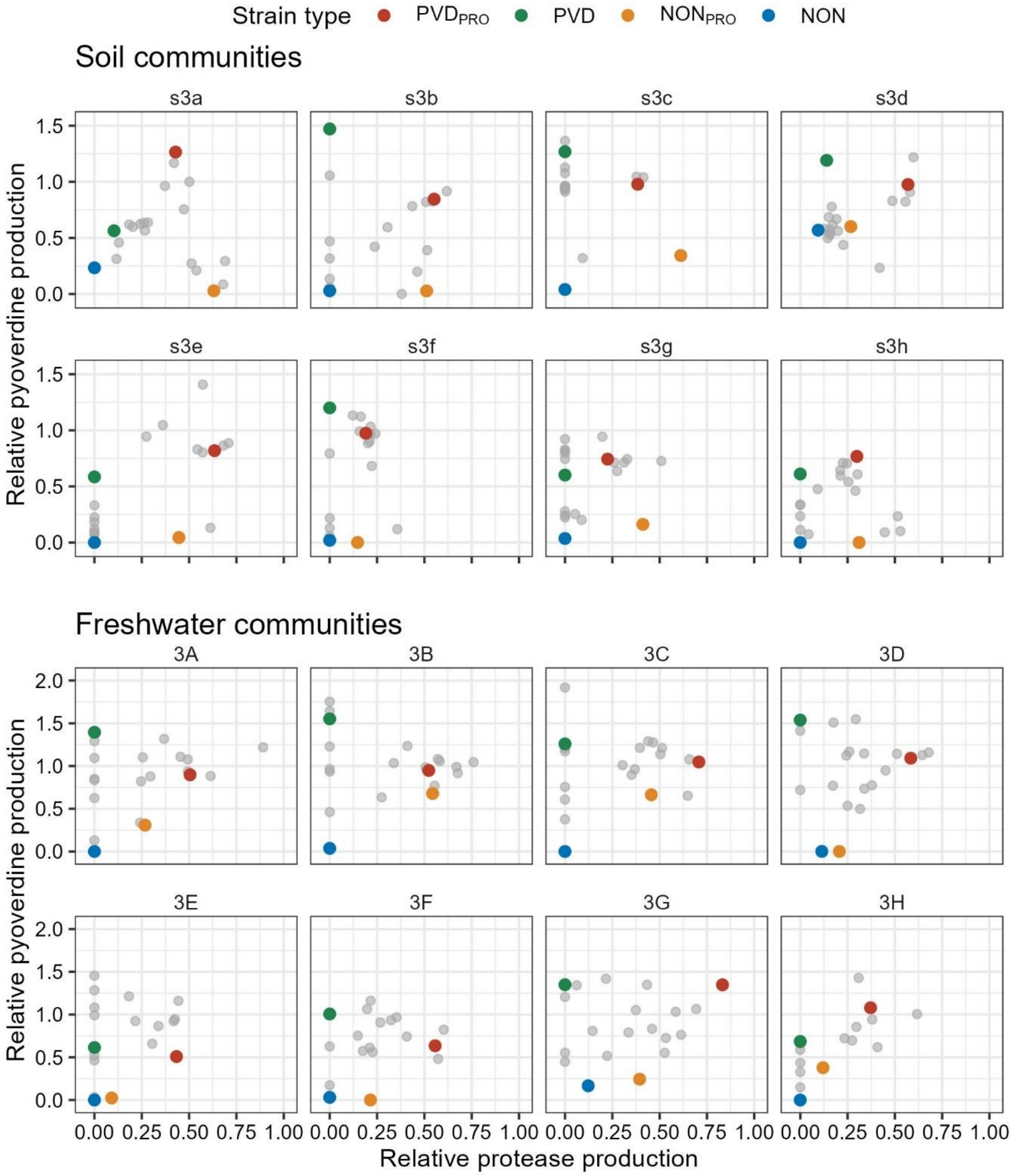

Figure S2 | Selection of strains belonging to four different types. We selected (i) one strain producing pyoverdine and proteases (PVD_{PRO}; red), (ii) one strain producing only pyoverdine (PVD; green), (iii) one strain producing only proteases (NON_{PRO}; orange), and (iv) one strain producing neither pyoverdine nor proteases (NON; blue) from each of 16 sets of 18-20 *Pseudomonas* strains from soil and freshwater samples. Where 'clean' phenotypes were not available, we picked strains similar to the intended type. The remaining (non-selected) strains (grey) are shown for comparison. Protease and pyoverdine production values are relative to those of the laboratory reference strain *P. aeruginosa* PAO1.

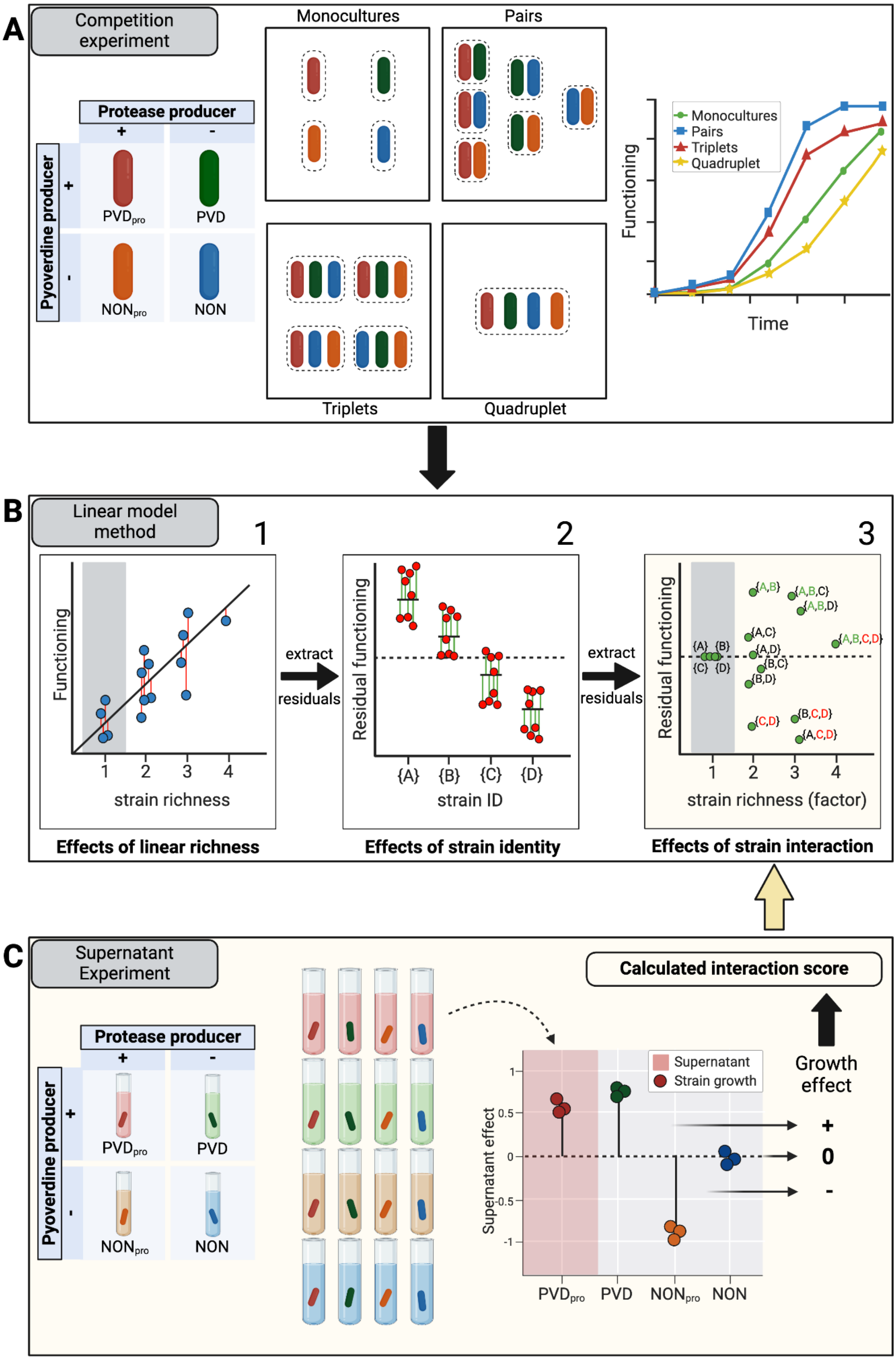

A
Competition experiment
Protease producer
+
-
Pyoverdine producer
+
-
PVDpro
PVD
NONpro
NON
Monocultures
Pairs
Triplets
Quadruplet
Functioning
Time
Monocultures
Pairs
Triplets
Quadruplet
B
Linear model method
1
2
3
Functioning
strain richness
extract residuals
Residual functioning
{A} {B} {C} {D}
strain ID
Residual functioning
strain richness (factor)
{A,B}
{A,B,C}
{A,B,D}
{A,C}
{A,D}
{B,C}
{B,D}
{A,B,C,D}
{C,D}
{B,C,D}
{A,C,D}
Effects of linear richness
Effects of strain identity
Effects of strain interaction
C
Supernatant Experiment
Protease producer
Pyoverdine producer
PVDpro
PVD
NONpro
NON
Calculated interaction score
Growth effect
Supernatant
Strain growth
Supernatant effect
1
0.5
0
-0.5
-1
PVDpro
PVD
NONpro
NON
+
0
-

Figure S3 |Graphical summary of the experimental and statistical approach. For each of 16 communities, we focused on iron-limited and iron-rich conditions and combined (A) competition experiments with (B) an established linear model method and (C) supernatant assays to unravel the effects of strain interactions and strain identity on community functioning. In particular we first grew four strains varying in their production of pyoverdine and proteases (PVD_{PRO}, PVD, NON_{PRO}, and NON) as monocultures and in all possible combinations of two, three, and four strains, and then quantified the cumulative growth and pyoverdine production of these cultures over time as our measures of community functioning (A). Next, we used a linear model method to partition variance in community functioning into effects of linear strain richness, strain identity, and strain interactions. The linear model method first accounts for effects of linear richness, which capture the extent to which functioning increases linearly with the number of strains in a community (B.1). Next, the method accounts for effects of strain identity on residual functioning (i.e., variance in functioning not explained by linear strain richness; red vertical lines in B.1) by examining whether specific strains growing in combinations with different community members consistently increase or decrease functioning (B.2). Finally, the method accounts for additional effects on residual functioning (i.e., variance in functioning neither explained by linear strain richness nor strain identity; green vertical lines in B.2) caused by strain interactions (B.3). To this end, strain richness is used as a categorical predictor to capture non-linear (non-additive) effects of strain richness, which then serve as a proxy measure for social interactions. For instance, (B.3) shows a hypothetical scenario wherein strains {A} and {B} engage in a mutualistic interaction, whereas strains {C} and {D} engage in interference competition, leading, respectively, to large positive and negative effects on residual functioning if (and only if) these strains grow together. Note that we did not consider data from monocultures in our linear models (strain richness = 1; grey horizontal rectangles in B.1 and B.3) because they do not feature strain interactions. To corroborate the validity of this statistical measure of strain interactions, we finally used supernatant assays to experimentally infer an interaction score (C). Specifically, we exposed each strain to supernatant from each of its community members, and then quantified the effects of the secreted compounds therein on the focal strain's growth (+, 0, or -, assigned values of 1, 0, and -1, respectively). Next, we calculated, for each possible combination of two, three, or four strains, an interaction score by averaging over the growth effects that the constituent strains had on each other. Finally, we integrated this interaction score into the linear model procedure to replace categorical strain richness as a measure of social interactions (yellow background of B.3 and C), which allowed us to compare the impact on functioning of our experimentally derived versus statistically derived measures of social interactions [Figure created in BioRender: Kuemmerli, R. (2025) https://BioRender.com/q40p772].

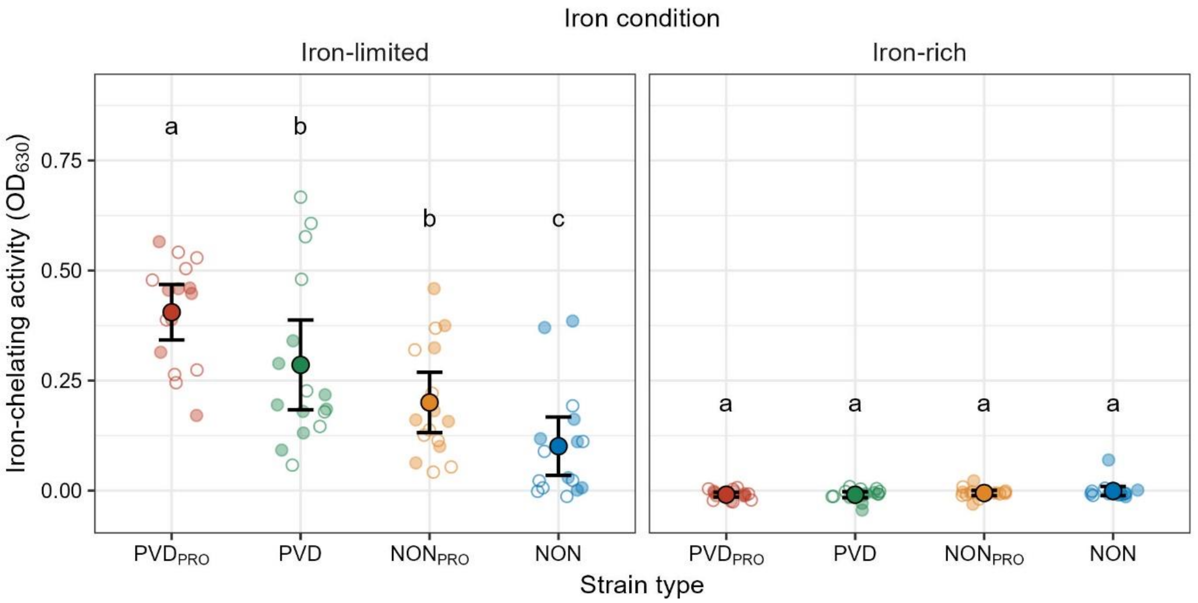

Figure S4 | Total iron-chelating activity of supernatant donors. Total iron-chelating activity of PVD_{PRO} (red), PVD (green), NON_{PRO} (orange), and NON (blue) strains isolated from eight soil (empty small circles) and eight freshwater (filled small circles) communities (one strain per type and community), measured in iron-limited and iron-rich medium. Large circles and black lines show mean and standard error, respectively. Small circles represent the median of four replicates obtained for each strain under each condition. Letters show significantly different types. All comparisons were performed within each medium (detailed statistical results are provided in Table S6).

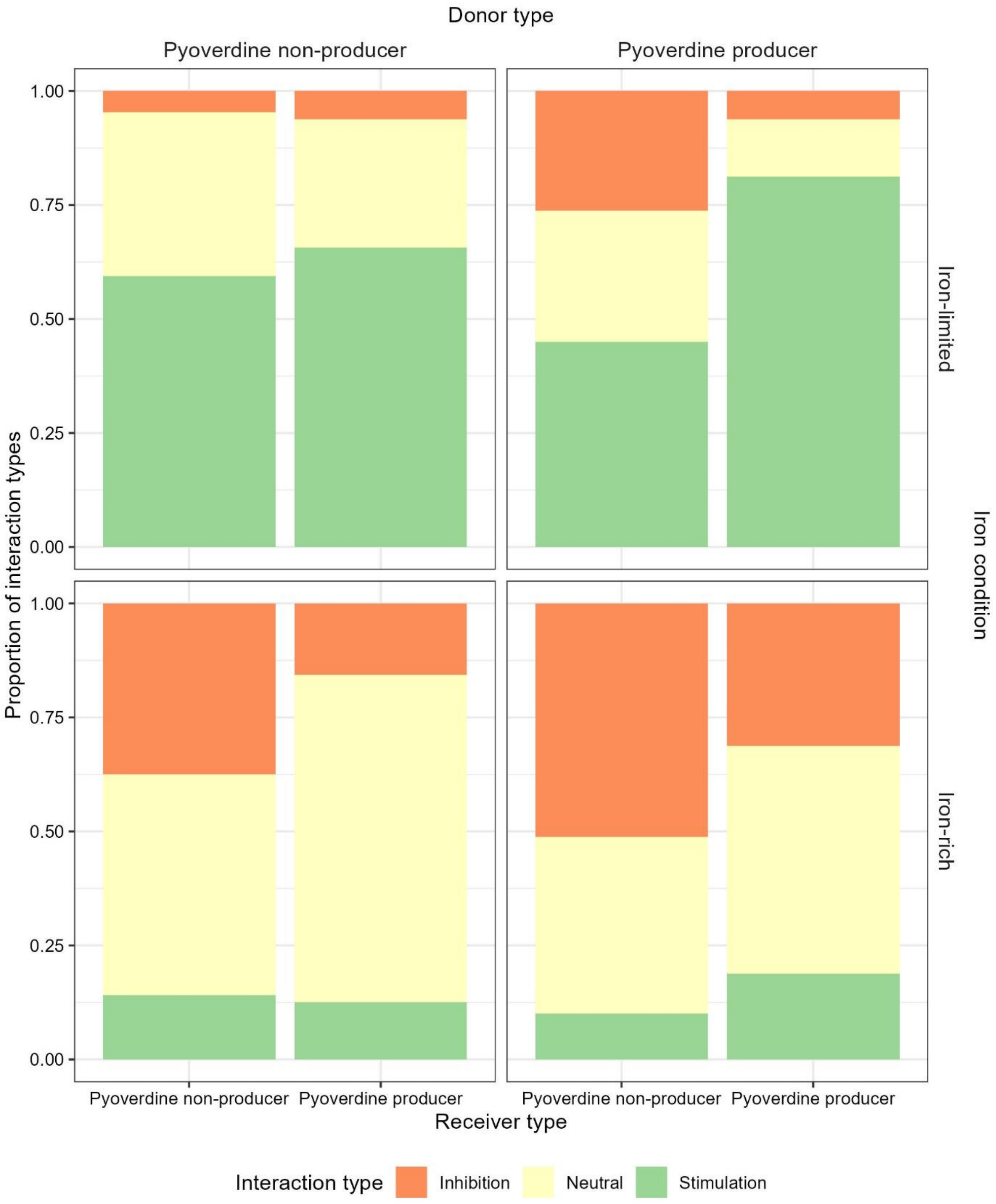

Figure S5 | Proportions of interaction types. Proportion of inhibitory (orange), neutral (yellow), and stimulatory (green) interactions between pyoverdine producers (PVD_{PRO} and PVD) and non-producers (NON_{PRO} and NON). Interactions were quantified under iron-rich and iron-limited conditions as supernatant effects mediated through compounds secreted by the donor and fed to the receiver. An in-depth analysis revealed that interaction types are jointly shaped by iron condition as well as receiver and donor type (see the Supplementary Analyses above for details).
